# To what extent can the activities of the South Australian Health in All Policies initiative be linked to population health outcomes using a program theory-based evaluation?

**DOI:** 10.1186/s12889-019-6408-y

**Published:** 2019-01-18

**Authors:** Fran Baum, Toni Delany-Crowe, Colin MacDougall, Helen van Eyk, Angela Lawless, Carmel Williams, Michael Marmot

**Affiliations:** 1 Society and Equity, Southgate Institute for Health, Flinders University, GPO Box 2100, Adelaide, 5001 Australia; 2 College of Medicine and Public Health, Flinders University, GPO Box 2100, Adelaide, 5001 Australia; 3 College of Nursing and Health Sciences, Flinders University, GPO Box 2100, Adelaide, 5001 Australia; 4 Health Determinants and Policy, SA Department for Health and Wellbeing, PO Box 6, Rundle Mall, Adelaide, 5000 Australia; 5 Department of Epidemiology and Public Health, University College London, 1-19 Torrington Place, London, WC1E 7HB UK

**Keywords:** Health in all policies, Social determinants of health, Intersectoral action, Healthy public policy

## Abstract

**Background:**

This paper reports on a five-year study using a theory-based program logic evaluation, and supporting survey and interview data to examine the extent to which the activites of the South Australian Health in All Policies initiative can be linked to population health outcomes.

**Methods:**

Mixed-methods data were collected between 2012 and 2016 in South Australia (144 semi-structured key informant interviews; two electronic surveys of public servants in 2013 (*n* = 435) and 2015 (*n* = 483); analysis of state government policy documents; and construction of a program logic model to shape assessment of the feasibility of attribution to population health outcomes).

**Results:**

Multiple actions on social determinants of health in a range of state government sectors were reported and most could be linked through a program logic model to making some contribution to future population health outcomes. Context strongly influences implementation; not all initiatives will be successful and experimentation is vital. Successful initiatives included HiAP influencing the urban planning department to be more concerned with the health impacts of planning decisions, and encouraging the environment department to be concerned with the health impacts of its work.

**Conclusions:**

The theory-based program logic suggests that SA HiAP facilitated improved population health through working with multiple government departments. Public servants came to appreciate how their sectors impact on health. Program logic is a mechanism to evaluate complex public health interventions in a way that takes account of political and economic contexts. SA HiAP was mainly successful in avoiding lifestyle drift in strategy. The initiative encouraged a range of state government departments to tackle conditions of daily living. The broader underpinning factors dictating the distribution of power, money and resources were not addressed by HiAP. This reflects HiAP’s use of a consensus model which was driven by (rather than drove) state priorities and sought ‘win-win’ strategies.

## Background

There have been many international calls for effective action on the social determinants of health (SDH) involving a range of government sectors [1–6]. Health in All Policies (HiAP) was developed by the European Union, and the WHO defined it as: ‘an approach to public policies across sectors that systematically takes into account the health implications of decisions, seeks synergies, and avoids harmful health impacts in order to improve population health and health equity’ [7]. HiAP initiatives have been adopted in many cities, regions and countries (see for example [1–3]). Establishing the health impact of HiAP is methodologically challenging. There are multiple problems with applying randomised control trials to complex entities such as cities, regional and national governments [4, 5, 8]. Foremost among the problems are the difficulties of establishing control units that are really identical, the complications of ‘controlling’ the activities of experimental or control administrative units, then making realistic claims about attribution of observed differences in health status in complex systems where many factors affect health and outcomes may take years or decades to become apparent. In response, evaluators have advocated the use of logic models based on chains of causality to assess impact [6, 9, 10]. Such models are particularly useful when combined with insights from the critical realist evaluation approaches. A central aspect of this form of evaluation is a concern with causality and the identification of causal mechanisms in social phenomena like a HiAP initative in a manner quite unlike the positivist search for causal generalizations [11]. Emphasis is placed on the contribution that a policy intervention makes to an outcome and views this contribution within its social, economic and political context [12]. These models map context, detail mechanisms that influence pathways to outcomes, and measure outputs and outcomes. Thus logic models using a critical realist approach hold most promise for untangling the question of whether HiAP is effective in promoting health [13, 14]. Our study is the first application of such models to HiAP to assess whether it has contributed to health.

### The context of South Australian HiAP

In 2007 the South Australian (SA) Government adopted HiAP, building on a long history of healthy public policy advocacy and innovation in South Australia [15]. By 2008 a dedicated unit had been established within the Health Department, and HiAP was established as a cross-government process formally endorsed by Cabinet. Governance for the initiative was linked to the State Strategic Plan, and an intersectoral ‘Health Lens Analysis’ process was implemented [16]. HiAP has operated continually since this time. SA HiAP involves multiple partners with sometimes divergent agendas implemented in, and influenced by, changing political, organisational and economic contexts, which are vital to understanding its implementation and impact [17].

South Australia’s population is 1.6 million with 1.2 million in its capital, Adelaide. From 2014 to 2016, South Australia has had a high life expectancy of 80.4 years for males and 84.5 years for females, increasing by 1.8 years (males) and 0.9 years (females) over 10 years [18]. There are, however, significant health inequities. In the period 2009–2011 Aboriginal and Torres Strait Islanders died 10 years before other Australians, and people in the lowest socioeconomic areas lived about 3 fewer years than those in the highest areas [19]. Over the study period (2012–2016) there were significant changes in the economic fortunes of the state that affected HiAP. Less favourable economic circumstances meant social priorities were subsumed by an economic focus leading to cost cutting in the public service. The HiAP initiative survived by aligning with the new mandates and ensuring it retained relevance to other sectors [20].

Implementation of HiAP used limited resources, including a small and varying number of staff (6 FTE at full complement). Staff costs in 2016 were approximately $550,000 pa. The SA Health Department budget for 2015–16 was $5.8 billion and HiAP totalled 0.00948% of that budget.

South Australia’s authorising environment at the time gave HiAP a mandate to work with other sectors on government priorities. The authorising environment included the government’s requirement for intersectoral collaboration to achieve the targets in South Australia’s Strategic Plan, and reporting requirements to Cabinet to ensure departmental accountability for achievement of these targets (discussed further below). While engaging with HiAP, other sectors conducted their normal business in an adapted way and some provided in-kind support and contributed limited additional resources.

This overview paper reports a five-year study using a theory-based program logic framework and addresses the question: To what extent can the activities of the South Australian Health in All Policies initiative be linked to population health outcomes using a program theory-based evaluation?

## Methods

We collected mixed-methods data (described below) within a theory-based program logic framework [12] over five years (2012–2016). This paper reports on the population health outcomes that, using the theory-based program logic framework, can be claimed to be likely from the SA Health in All Policies intiative. It draws on new data analysis as well as prior data analyses from the evaluation that have been reported in other papers [20–23]. The other papers consider HiAP processes and show how we have developed and applied the theory-based logic model to understand HiAP’s influence on population health outcomes. These papers and their key findings are summarised in Table 1. Drawing on these prior papers in addition to new analyses undertaken for the current paper is vital because the evaluation has produced considerable amounts of data that cannot be adequately presented and discussed within the contraints of a single overview paper.

**Table 1:** Summary of key findings from published papers

Paper published from the research	Key findings
Evaluation of Health in All Policies: concept, theory and application [8]	- Developed through a consultative process and informed by social and political science theory, program logic can accommodate the complexity of public policy-making.
Developing a framework for a program theory-based approach to evaluating policy processes and outcomes: Health in All Policies in South Australia [12]	- Program logic and its underlying theory of change provide a framework within which attribution of health outcomes can be made through a predictive chain-of-logic approach.
	- The program logic framework provides a basis to explore interactions between the framework’s components and how they shape policy-making and public policy.
	- Using a program logic framework allowed for assessment of HiAP’s success in integrating health and equity considerations in policies and laid the foundations for predicting the impacts of resulting policies.
Creating a burden of evidence to consider the impact of Health in All Policies: A program logic approach [42]	- A case study of a HiAP project undertaken with Education Department staff to increase parental engagement in child literacy is used to show how the research built a ‘burden of evidence’ that supports logically coherent chains of relationships between HiAP activities and intended outcomes, which have been explained through a program logic model.
Health in All Policies in South Australia: what has supported early implementation? [22]	- Implementation of the SA HiAP approach was supported by dedicated staff and adequate financial resources; a central mandate that created an authorising environment and supported the entry of HiAP staff into other government departments; alignment of HiAP with government core business and strategic priorities; and establishment and maintenance of trust in, and credibility of, the HiAP approach and staff.
	- Relationship development and maintenance was central, and a focus on co-benefits supported development of these relationships.
	- Dominance of siloed government structures and decision making and narrow definitions of core business threaten HiAP success and reduce its acceptance.
Ideas, actors and institutions: Lessons from South Australian Health in All Policies on what encourages other sectors’ involvement [20]	- Wide acceptance among participants of role of social determinants in shaping health and of importance of action to promote health in all participating government agencies.
	- The existence of a HiAP Unit helped gain support from other sectors.
	- Other sectors became involved in HiAP because of the presence of a supportive, knowledgeable policy network of public servants, a clear political mandate, a move from a short term project focus to institutionalisation through new public health legislation, and finding a fit between HiAP ideas and the dominant economic paradigm of government.
	- Policy entrepreneurs and champions played a critical role in supporting and disseminating understanding of healthy public policy and social determinants of health.
Health in All Policies in South Australia—Did It Promote and Enact an Equity Perspective? [21]	- The SA HiAP approach had dual goals of facilitating joined-up government for co-benefits, and addressing social determinants of health and inequities through cross-sectoral policy activity.
	- Government agencies understood HiAP as a catalyst for collaboration, and as providing tools for improving intersectoral policy development, but did not understand HiAP’s equity goal, which gained little traction.
	- Where equity is not seen as core government business, it can be viewed by agencies as optional and can struggle to achieve prioritisation against competing political agendas.
	- HiAP’s co-benefits approach has been central to the SA HiAP approach and brought significant benefits to participants from other sectors. The goal of establishing and maintaining relationships for co-benefits was privileged over equity outcomes, so that equity became practically invisible in HiAP activity.
	- HiAP’s initial intentions to address equity were only partially enacted and little was done to reduce inequities.
Understanding Australian policies on public health using social and political science theories: reflections from an Academy of the Social Sciences in Australia Workshop [43]	- Most governments do not prioritise action on social determinants of health and health equity.
	- Applying multiple theories is helpful in directing attention to, and understanding, the influences of the different stages of the policy process. The application of theory promises to be most effective when it is multidisciplinary and blends and applies insights from a number of different theories.
	- There is value in collaboration between public health researchers, political and social scientists and public servants to open up critical discussion about the intersections between theory, research evidence and practice.
	- Critique is vital to make visible the processes through which some sources of knowledge may be privileged over others, and to examine how political and bureaucratic environments shape policy proposals and implementation.
Health Impact Assessment in New South Wales & Health in All Policies in South Australia: differences, similarities, and connections [23]	- Health impact assessment (HIA) and HiAP approaches have similar overall intents to facilitate engagement of other sectors to consider the health implications of their policies.
	- Key differences are in underpinning principles, technical processes and tactical strategies, which appear to stem largely from organisational positioning of the work and the extent of links to government systems.
	- Alignment of the HiAP approach with government systems increases its likelihood of influence in the policy cycle but political priorities and government sensitivities can limit the scope of HiAP work. Implementation of the HIA approach from outside government gives greater freedom to collaborate with different partners and assess priorities without the constraint of government priorities. However, greater distance may also reduce the potential impact on government policy.
New norms new policies: Did the Adelaide Thinkers in Residence scheme encourage new thinking about promoting wellbeing and Health in All Policies? [44]	- The Adelaide Thinker in Residence scheme was an innovative program to encourage a more flexible, responsive and adaptable SA public sector, through expert international Thinkers introducing new strategic ideas to address complex problems. It highlighted the need for intersectoral collaboration and a mutually reinforcing agenda across government to advance a social determinants approach.
	- As external entrepreneurs, the Thinkers built on the work of local entrepreneur networks to advance their policy agendas, including presenting prevention as important to economic goals.
	- The scheme enhanced commitment to public health and health promotion, and highlighted the importance of investing in disease prevention and health promotion, including through addressing social determinants outside the health sector.
	- By strengthening and recasting norms and establishing a stronger and more extensive policy network, a tipping point was reached for the adoption of new norms within the bureaucracy. Intersectoral networks were mobilised, and the issue of health was expanded to one of economics and governance, thus increasing likelihood of institutionalisation.
	- A HiAP approach was proposed through this scheme, with health reframed as an economic concern. HiAP was directly linked to the government’s broader political priorities, which supported its implementation in SA.

*Abbreviations*: *HiAP* Health in All Policies, *HIA* Health impact assessment, *SA* South Australia

The methods are summarised in Table 2 and elaborated on below and have been described previously [20].

**Table 2:** Methods

Method	Description
Collaborative development of program logic model (PLM)	PLM developed with policy actors and sets out the theory behind HiAP and mechanisms – ‘the underlying entities, processes or structures’ that contribute to outcomes. The critical realist PLM provided a frame for the evaluation by mapping context, details of the HiAP mechanisms and the anticipated outputs and outcomes.
Semi-structured interviews with policy actors	Between January 2013 and June 2016 144 semi-structured interviews were undertaken. 53 of the interviews were with staff from the SA Health Department, 51 involved staff from other sectors of the SA State Government, 31 involved staff from local government, five involved academics who had knowledge and experience of the HiAP initiative and four involved politicians or political staff. Interviews related to specific projects (*n* = 39) and the overall HiAP initiative (*n* = 105).
Electronic survey of public servants	An online survey of public servants was conducted in 2013 and repeated in 2015. Individual public servants in the HiAP policy network were identified with the assistance of HiAP staff and comprised public servants who had had contact with HiAP since 2007. In 2013 and 2015, the policy network involved 435 and 483 public servants respectively (for further details see [22]).
Analysis of state government policy documents	Ongoing during research period to track changes in state priorities.

*Abbreviations*: *HiAP* Health in All Policies, *PLM* Program Logic Model, *SA* South Australia

### Development of a program logic model

**Fig. 1:**
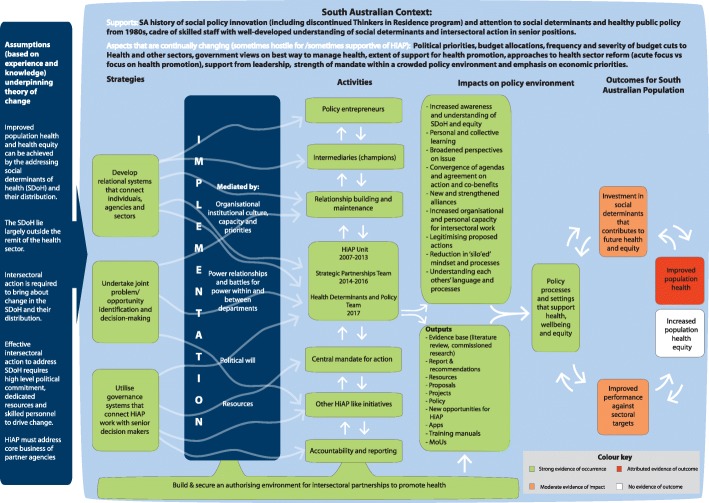
South Australian Health in All Policies initiative program logic

At the outset of the research, a collaborative process was used to develop a theory-based program logic model (PLM) as part of our theory-based evaluation framework from which the assessment of health impact could be made (for details of the framework and process of developing it see [8, 12]). The model in Fig. 1 outlines the program theory underpinning HiAP and applies contribution analysis to determine the extent to which the activities and strategies of HiAP can be argued to have contributed to improved health. The strength of evidence we have to support our claims in relation to the potential for SA HiAP to achieve health outcomes in each part of the model in Fig. 1 is indicated with green shading equating with strong evidence, orange indicating moderate, and red indicating evidence relying on a projected contribution, based on the body of existing evidence indicating what health outcomes are likely to flow from particular actions.

### Semi-structured interviews with public servants

Between January 2013 and June 2016 144 semi-structured interviews were undertaken. 53 of the interviews were with staff from the SA Health Department, and 51 involved staff from 15 other departments/agencies of the SA State Government who had been involved with the HiAP approach between 2007 and 2016. The 15 other departments/agencies included: community services, education, justice, transport, governance, infrastructure, employment, trade, and natural resources. Five of the interviews involved academics who had knowledge and experience of the HiAP initiative and four involved politicians or political staff (Table 2).

The evaluation was informed by the application of social and political science theory so that interview questions considered, for example, agenda setting, the role of actors as champions in diffusing HiAP ideas, and why and how particular HiAP activities and outputs lead to distal health outcomes (further details are available at [12]). The later interview schedules were adapted in light of emerging findings from the earlier interviews and the survey, so that for instance in the later interviews the impact of the worsening economic situation in South Australia on the public service was included.

Six of the research team, all very experienced in qualitative interviewing, conducted the interviews using the pre-prepared interview schedules which, in relation to outcomes, sought interviewee’s views about key markers of success of the SA HiAP approach, the extent to which understandings about health and wellbeing have been incorporated into participants’ thinking and other departments’ core business, and the extent to which South Australia benefited from its involvement with HiAP. The interviews were recorded and transcribed verbatim for all but two respondents. The interviews averaged 38 min, ranging from 10 min to 1 h 35 min. Interviewees were able to review their transcript and seven chose to check and amend their transcript by either editing the transcript directly or sending email notes to clarify or elaborate on a particular aspect of the interview. Two people requested written notes be taken by the interviewer rather than a recording. In these cases the notes were checked by the interviewee.

### Electronic survey of public servants

An online survey of the HiAP policy network in the SA public sector was conducted in 2013 and repeated in 2015. HiAP staff assisted the research team to identify public servants who had had some contact with HiAP since 2007. In 2013 and 2015 the network involved 435 and 483 public servants respectively. The survey samples for 2013 and 2015 were selected from the identified public servants and included only people who were working within the SA Government at the time of each survey. In 2013, 373 public servants were invited via email to participate in the first survey. 168 (45%) of these people provided meaningful responses by answering survey questions beyond the initial demographic questions. Six (2%) people answered only the demographic questions and their responses were excluded from the analysis. 199 (53%) people did not respond at all. In 2015, 339 people were invited via email to participate in the second survey. 151 (45%) of these people provided meaningful responses by answering survey questions beyond the initial demographic questions. 25 (7%) people answered only the demographic questions and their responses were excluded from the analysis. 163 (48%) people did not respond at all. Each potential respondent was contacted four times [24]. The surveys sought information about respondents’ awareness of the HiAP approach, their experiences of collaborating with HiAP, and their perceptions of the outcomes of HiAP work. The survey data are used in this paper to report on the perceptions of public servants on the impact of HiAP, on their understanding of social determinants, and the impact that the work of their department has on population health.

### Case studies

The research included case studies of the major work conducted by the HiAP team either as Health Lens Analysis projects or subsequently as part of other government initiatives (further details of these case studies are available at [21]). These case studies have been used to frame the case for the contribution analysis and attribution to projected population health outcomes.

The criteria we used to select the Health Lens Analysis (HLA) projects (discussed in more detail below) as case studies were: a) had progressed sufficiently to allow assessment of what outputs had been produced; b) involved a number of different departments to ensure collection of a range of sectoral views; and c) incorporated a focus on Aboriginal and Torres Strait Islanders as well as other population groups. During selection we ensured that we did not select HLA projects that were led by departments that were undergoing severe cuts or restructures. Securing interviews in such departments would have been logistically and politically difficult. Furthermore, we selected some case studies that followed the usual HLA process, and others in which a modified process had been applied to manage decentralised decision making processes and/or alternative managerial structures. Case studies were selected progressively as the research project continued. This allowed us to select some examples of HLAs that had been initiated to meet South Australia’s Strategic Plan targets, as well as some that had been initiated to fulfil the requirements of the newer legislative driver, the SA Public Health Act.

While there may have been potential for the findings from the multiple datasets to be conflicting, we found that rather they built upon each other and were used iteratively in our analysis. Semi-structured interviews were undertaken progressively throughout the research – before, during and after the collection of data from other methods. Follow up interviews were undertaken with key individuals to understand the changing contextual factors that were identified during the surveys, as well as the developments in particular case studies that were identified during the document analysis. Interview data also informed the development of survey questions and guided examination of case study documents. The surveys provided a greater breadth of data than the interviews, which involved deep examination of particular informants’ experiences. As a result of this research approach, each method supported and informed the other.

### Analysis of data

A collaborative thematic analysis of the interview transcripts was conducted using the qualitative analysis software NVivo 11. The initial round of open coding involved two members of the team reading the transcripts to identify central themes followed by collaborative coding sessions with other investigators. Selective coding was applied to examine respondents’ discussion of key outputs and outcomes. During the selective coding process, we used the program logic model [12] to identify and organise the data against the three most relevant components of the model that link to the anticipated program outcomes: outputs, impacts on the policy environment, and policy processes and settings supportive of health, wellbeing and equity. Numerical data from the survey were analysed using SPSS software. Cross tabulations compared relationships between responses to selected questions.

Throughout the research, emerging findings were fed back to participants and informed the ongoing implementation of HiAP. For the duration of the research, the program logic framework provided a means to track changes to HiAP and the program theory inherent within it [12].

All data collection activities received prior approval from the Flinders University Social and Behavioural Research Ethics Committee and the SA Health Human Research Ethics Committee. Informed consent was provided by all participants in the research prior to their interview.

## Results

Our findings are shaped by the program logic model (see Fig. 1) and describe how HiAP encouraged action on social determinants, and the likelihood that these actions have led or will lead to improved health and equity.

### Action on the social determinants of health

The program theory held that improved health would result from intersectoral policy development to encourage action on the SDH. From 2009 to 2013 HiAP used Health Lens Analysis (HLA) to work with other sectors to determine what action on SDH was required [25]. HLA is a collaborative process between health and another sector to examine the health impacts of a given policy area [8]. The HLAs were effective in highlighting relevant SDH and were broadly supported by policy actors from the other sectors [20].

Table 3 presents an analysis of the program logic strategies relating to the main HiAP activities in Fig. 1:

- Utilisation of governance systems to support SDH and equity
- Development of relational systems to support action on SDH
- Joint problem identification and decision making between HiAP and other sectors

**Table 3:** Reflection of aspects of program logic in SA HiAP action on the social determinants of health (2007–2016)

Focus of action or intent	Approach	Reach	Program theory: Immediate & Intermediate Outcomes	Health and equity outcome contribution and likelihood
Utilisation of governance systems to support social determinants of health and equity				
South Australia’s Strategic Plan (SASP) provided medium to long term goals for SA in identified priority areas	SASP provided strong authorising environment for HiAP	Whole population of SA	A clear plan for improving the population’s health and wellbeing	If government focus on achieving SASP targets maintained, likely to contribute to positive health and equity outcomes
	HiAP initial activity focused on working with different agencies on health impacts of relevant SASP targets, many becoming focus of HLA work			
	Departmental Chief Executives held accountable for aspects of the plan, encouraging them to collaborate with HiAP			
Assessment of extent to which SDH are presented in SA’s 7 Strategic Priorities for SA’s future	HLA of SA’s 7 Strategic Priorities	Reach varies depending on priority, but overall, whole of population	Shorter term focus than that of SASP on strategic areas of significant government attention	Enhanced whole of government responses to SDH
Legislative support for action on SDH through new Public Health Act	HiAP reflected as a principle in the new legislation, and systematised in local government-led regional public health planning and establishment of Public Health Partner Authority (PHPA) agreements with interested government departments and other organisations	Potentially whole of public sector and non-government organisations	The PHPA provide basis for on-going action on SDH by gaining commitment from organisations to undertake such action	Strong basis for improving health in the long term
Develop a statement to describe SA as a State of Wellbeing 2016	90 Day project – State of Wellbeing statement. Statement cites HiAP principles as underpinning statement and notes that wellbeing is most evident in equitable jurisdictions	Whole of population	Establishes aspiration for SA to be State of Wellbeing and positions equity as important to high wellbeing, which will encourage action across the state to advance wellbeing and equity	The aspiration for wellbeing and as part of this equity to be a goal of state policy could, if enacted, result in improved health and equity
Development of relational systems to support action on social determinants of health				
Changing culture of the SA public service to work collaboratively across sectors on social determinants of health	Changing culture of public sector to understand role of social determinants and importance of cross-sector work	Whole of public service	Improved joined-up policy responses to complex multi-sectoral issues	In long term public sector action more likely to work across silos and promote health (equity did not feature strongly)
			Ideas about SDH have been dispersed across the public sector and are encouraging more focus on the broader implications of different agencies’ business (including the health implications) in some sectors	
	90 day project on Joined-Up policy delivery (2016)			
	Many departments have nominated policy champions to work on joined up policy through a policy network			
Joint problem identification and decision making between HiAP and other sectors				
Improving early literacy in children through increased parental engagement	HLA on Parental Engagement in Literacy	Initially 4 selected schools, then contributed to strategy for entire Education Dept.	Improved literacy has been shown to have a positive impact on health	If children more literate then they will have improved health when they are adults [27, 45]
Identifying strategies to improve the health, sustainability and economic positioning of communities in Upper Spencer Gulf	HLA on Healthy Sustainable Regional Development: Upper Spencer Gulf	Regional community of 53,000 people	Including information about health and wellbeing in a regional plan will contribute to increased awareness in region and lead to improved policies	Slight evidence of impact on region and likely to be very minor
	Modest output of report and social health atlas and evidence of increased awareness from public servants			
Improve the health and wellbeing of international students	HLA on international student health and wellbeing	International students in SA (more than 20,000 attracted to higher education institutions each year, however focus of HLA was on a sub-set of these, in Vocational Education and Training sector)	Increasing students’ knowledge of Australian health system will enable them to gain care and stay healthy, but evidence on effectiveness of provision of information alone is not strong so unlikely to have big impact without more structural change [46]. For example, focus of information in fact sheets on general health and wellbeing is on individual behaviours such as smoking, alcohol, drugs, healthy diet etc.	Very minor
	The early stages of HLA suggested more wide-ranging outcomes whereas student fact sheets (reprinted and used interstate) focused mainly on behaviours, health insurance and available services			
Support vulnerable young people to successfully transition from education to further training and employment	HLA on Learning or Earning		No impact	N/A – did not progress
	Did not progress because health reorganisation slowed progress to extent that higher education dept. withdrew involvement			
Increasing the proportion of the population in healthy weight range	HLA on Healthy Weight which examined the potential contribution of every government dept. to encouraging healthy weight	Whole of population	Increase in healthy weight of population	Improved population health outcomes [47, 48]
Increasing Aboriginal life expectancy by improving road safety through increasing safe mobility options	HLA on Aboriginal Road Safety	Aboriginal people in SA	Increased number of Aboriginal people in urban, rural and remote SA who obtain and retain drivers’ licences	Reduce number of Aboriginal people killed in motor vehicle accidents
	HLA work informed 90 Day Project to frame Aboriginal drivers’ licensing as part of Public Sector Renewal Program, focused specifically on remote area with exclusively Aboriginal population			
				Reduce number of Aboriginal people incarcerated for traffic offences
				Increase mobility, improved road safety and ultimately improved health and wellbeing [49, 50]
Making Adelaide a less carbon-dependent city and increasing healthy transport options	3 HLAs on:	Detailed plans relevant to metropolitan region, and the active transport and cycling strategies relevant to whole Adelaide metropolitan area	Denser cities are more carbon efficient and so healthier	Health benefits likely over the long term from less carbon, more sustainable city and transport, and more active population [51, 52]
	Transit orientated developments			
			Active transport reduces carbon and increases human activity, and encourages social interaction which is also good for health	
	Active transport			
	Cycling			
Increase the proportion of the community who visit parks, and increase the regularity of park visits	Environment sector’s action under PHPA agreement on ‘Healthy Parks Healthy People’ with Health has led to strong focus on mental health and wellbeing impacts. Includes increasing green infrastructure in city.	Whole of population and state, including special focus on Aboriginal people	More people across all population groups use parks more often for a variety of activities	If promotion of use of parks progresses as planned is likely to lead to improved mental and physical health outcomes as people get the benefits of exercise, social interaction and being in nature [33, 34]
Encouraging action on the social determinants of health in local government	Local government Regional Public Health Plans mandated by SA Public Health Act 2011	Whole of state	Immediate outcome is that all local governments in SA have had to produce public health plans and so focus on how they will contribute to health in the future. This may lead to improved infrastructure for health throughout the state Local government reports that the process was useful and their plans will be more extensive in subsequent iterations	If the planning processes are effective and then implemented, health and wellbeing will be improved
	Detailed analysis of plans indicates a fit between them and HiAP principles articulated in 2007			
	Extent of new action limited in first round but promising			

*Abbreviations: dept*. department, *HiAP* Health in All Policies, *HLA* Health Lens Analysis, *PHPA* Public Health Partner Authority, *SA* South Australia, *SASP* South Australia’s Strategic Plan, *SDH* social determinants of health

Table 3 shows immediate and intermediate outcomes for each area and the longer-term likelihood of health outcomes.

### Utilisation of governance systems to support SDH and equity

***South Australia’s Strategic Plan*** (SASP) provided an initial authorising environment for HiAP. It included targets related to the SDH which departmental chief executives were accountable for achieving. This increased the chief executives’ motivation to co-operate with HiAP and provided a clear commitment to improving health and wellbeing.

In 2011, the SASP was supplemented by ***seven strategic priorities*** and HiAP led an HLA to determine how they related to health, for example exploring the relationships between the strategic priority ‘growing advanced manufacturing’ and population health and wellbeing, including through economic development, employment opportunities, and development of the advanced health-manufacturing sector. The health logic was that increased employment and economic development would have a positive health impact. In 2016 the government committed to making South Australia ***A State of Wellbeing*** and HiAP was central to the development of an intersectoral wellbeing statement which incorporated some SDH elements.

***A new Public Health Act*** reflecting new public health principles provided an additional authorising environment for HiAP and the legislative mandate for two important developments: Public Health Partner Authorities (PHPA) and Regional Public Health Plans (see Table 3 for examples).

### Development of relational systems to support action on SDH

#### Culture change in the SA public service

HiAP achieved some success in bringing about culture change in the public service whereby public servants in a wide range of departments came to appreciate that their work had an impact on population health and wellbeing, and that maximising the positive health impact was part of their role. Data from the qualitative survey and interviews with public servants suggest HiAP increased public servants’ awareness of the health impacts of their agencies’ policies. In the 2015 survey 55.4% of respondents reported that they could see clear links between the work of their department and the SDH, with 53.2% of respondents either agreeing or strongly agreeing that collaborating with HiAP had increased their understanding of the SDH and equity. The interviews indicated that HiAP had built networks, created synergies and broken down silos in the public service. Executives from transport, urban planning, education and infrastructure stressed that health was now routinely considered in their planning processes. For urban planning staff, this meant that the health staff were invited to comment on their revised planning legislation and health considerations were built into both the legislation and plans. Planning staff also reported “heightened recognition” of the importance of neighbourhood design to health, and health was considered in the planning of transit-oriented developments more than before HiAP’s involvement. Education staff reported that the health impact of their core work was more widely appreciated. Strength of attribution to long term outcomes can be seen in the traffic light shading in Fig. 1.

Public servants also noted that HiAP initiatives were “on a convergent path” with the agendas of their departments. A health public servant noted that a department “might have been thinking about it [a health focus] or working on it, considering it”, and HiAP had “given a leverage point to make it across the line”. An example was where the urban planning goal of creating a ‘vibrant’ city converged with the health goal of encouraging cycling for health benefits as cycling makes the city more ‘alive’. The Department of the Premier and Cabinet saw HiAP as one of the tools that produced coherent policy.

Public servants noted that when they changed work roles they continued to apply knowledge gained through involvement in HiAP. One survey respondent noted that in their new role “the understandings and benefits of this approach remain with me”. A departmental head made a similar comment about using her new position to embed health thinking.

#### Joint problem identification and decision making between HiAP and other sectors

Initiatives in this section are concerned with intersectoral work to address an area of government activity which affects SDH. The HiAP team sought to identify and negotiate HLA projects within the core business of the partner agency that were supported by research evidence for potential positive health outcomes.

#### Employment and education

Four HiAP projects directly addressed the two vital SDH of employment and education. A program to encourage parental engagement in children’s literacy was trialled at four schools. Public servants from Education in part attributed to HiAP the adoption of a focus on parental engagement in the revised Numeracy and Literacy (birth to 18) Strategy [26]. The well-established link between literacy and health supports a conclusion that increased literacy skills as children grow up would improve health [27, 28].

One HLA concerned the health and wellbeing of international students. Higher education is an important part of the SA economy. A report into overseas students’ experience in Adelaide had concluded that students had very little understanding of the Australian health system [29]. Our analysis indicated that while significant issues concerning SDH for international students were raised, the output from the project was a guide to the Australian health system for these students. The department responsible for higher education reported finding the HLA process overly complicated and prolonged, and a senior executive from that sector commented that “the reality is that we could do it on our own anyway and we could actually probably do it more efficiently and see those returns and the benefits actually a lot quicker”. In this case HiAP started with a more ambitious project concerning sexual health issues and safety for overseas students but the process of collaboration whittled the focus of the project down to a much less ambitious information pamphlet.

In terms of employment, HiAP’s involvement in planning for the development of a regional area of SA that was expecting a mining boom encouraged public servants to focus on the health and wellbeing aspects of the plan by producing a social health atlas of the region. Although the mining boom did not eventuate, public servants from the industry sector reported that HiAP involvement slightly increased awareness of SDH and laid the basis for future joint work which has eventuated. Another employment-focussed project with a different sector did not proceed, and our analysis indicates this was because the department concerned was beset with budget cuts and staff losses, and previous involvement with HiAP was perceived to have few benefits. Overall given the economic headwinds faced by SA it would have been hard for HiAP to make much contribution in an increasingly adverse employment environment in which casualization was increasing and unemployment rising [30].

#### Sustainable urban and regional planning and infrastructures

Some HiAP HLAs were concerned with planning and sustainability (see Table 3). Each showed explicit causal pathways between HiAP processes and likely future health outcomes. HiAP contributed to the state’s aim of making the capital Adelaide less carbon dependent, including increasing active forms of transport. Initially this HiAP contribution was through three HLAs on transit-oriented developments. The link to health (summarised in [31, 32]) is that a more carbon efficient economy would contribute to a sustainable environment, and active transport options would mean more people exercise and benefit from casual social interactions. Our study indicates that HiAP played an important role linking environmental sustainability directly to health outcomes supporting policy coherence. For instance an executive in the industry sector spoke of introducing health indicators into consideration of sustainable mining development to develop healthy communities.

The relational aspects of HiAP were reinforced following implementation of the Public Health Act which provides a legislative mandate for intersectoral action. The Act established Public Health Partner Authorities (PHPA) whereby agencies could sign a formal agreement with the Department of Health concerning action on social determinants. One example was the PHPA agreement between health and the environment sector on Healthy Parks, Healthy People. This agreement provided a legislated institutional framework for co-benefits to be achieved by health and the environment sectors including helping to secure longer term population health and wellbeing outcomes as a result of increased green space in which people can spend time [33, 34]. This initiative is a whole-of-population initiative with a special focus on Aboriginal health, and has the potential to reduce inequities over the longer term. The strength of attribution can be seen in the traffic light shading in Fig. 1. The PHPA agreements provide instrumental support for the relational work of HiAP and made a direct impact on the policy environment by supporting converging agendas, strengthened alliances and organisational capacity for intersectoral work. Finally, our analysis showed that many of the 2007 HiAP principles were reflected in SA local government’s Regional Public Health Plans. These plans partly reported existing actions by councils to address the SDH and their intentions to expand their actions.

#### Healthy weight

The SASP set the goal of increasing the percentage of the state’s population at a healthy weight. The need for such a goal is evident – in 2014–15, 65.8% of South Australians over 18 were either overweight or obese, increasing from 50% in 2004–05 [35]. HiAP commissioned work to determine what government departments were doing and could do to contribute to achieving the goal. Many actions were identified including creating community vegetable gardens in public housing areas, intensifying active transport initiatives, increasing visits to parks, and increasing healthy food supply in prisons [36].

#### Aboriginal drivers’ licensing

Aboriginal drivers’ licensing was a multi-sectoral project with initially wide-ranging aims that were finally reduced to interventions in one remote Aboriginal community in the state. As a direct result of HiAP, legislative and policy changes were made to make the licensing system fairer for Aboriginal people living in this community. HiAP was the first of multiple initiatives addressing this issue, making attribution difficult. However, HiAP evidence gathering and recommendations did influence the work being undertaken. The eventual changes increased driver training for some Aboriginal people, so that road injury and deaths can be expected to be avoided in the long term and fewer Aboriginal people are likely to be imprisoned for licence infringements.

#### Co-benefits approach

The selection of HLA projects was based on a co-benefits approach, requiring the mutually agreed identification of projects that would address the objectives of the participating partner agency while having potential health outcomes according to HiAP’s research evidence. While the focus of the topics negotiated between HiAP and their partner agencies was clearly the core business of the partner agencies (eg a child literacy project with the Education Department, Aboriginal road safety with the Transport Department, and increasing green space with the Environment Department), the involvement of HiAP ensured a new and more critical focus on the health implications of policy decisions in partner agencies’ consideration of their core business. This enabled other sectors to develop understanding of their role in addressing the social determinants of health, resulting in increasing coherence across government policies and an increased awareness among participants of the broader health and wellbeing implications of their agencies’ policies for the SA community.

## Discussion

Our main finding is that, through using a pragmatic and theory-based program logic approach to causal analysis, SA HiAP can be judged to have made a modest contribution to actions likely to have improved population health in South Australia. It did this using the activities and policy processes shown in Fig. 1. The intiative was small: the SA Health budget for 2015–16 was $5.8 billion. HiAP totalled 0.00948% of that budget. Judged against this modest input, impact in terms of changing the culture of the public service and encouraging health to be understood as an important consideration in other policy areas, especially in urban planning and the environment, was significant. We argue that most actions listed in Table 3 are likely to lead to longer term health benefits given that the evidence links these interventions to health outcomes. Of course whether those health outcomes eventuate will also depend on the South Australian and global context. Contextual factors reducing the support from other departments accounted for one initiative not proceeding. In another instance we observed lifestyle drift from an initially wide-ranging identification of social wellbeing concerns for overseas students to an information pamphlet.

The activities and impacts shown in green in Fig. 1 were found to have occurred and collectively to have built an authorising environment for a range of policy processes that support health and wellbeing. Our evidence indicates that HiAP helped public servants to appreciate how their sectors affected health [20]. We predict this will be positive for the state’s future but also caution that the government’s response to the economic climate by reducing the public service results in staff turnover, loss of capacity and knowledge. This may have a negative impact, outweighing the positive HiAP effect. Elsewhere we have noted that the pursuit of health equity was more prominent in the early days of HiAP where some rhetorical support existed for progressing an equity agenda [21]. Subsequently, however, economic pressures resulted in the government narrowing its priorities to economic goals, and reflecting HiAP’s dependency on its political context, little was done to address inequities in our study period.

A major strength of our study is that it is the only application of a theory-based program logic to assess the likely contribution of a complex, longer term public health initiative. While we can only make limited attributions of causality of HiAP to improved health and wellbeing in South Australia, we can do this within a predictive model which sets the likely outcomes and the processes by which they are achieved [12]. The use of program logic to support a narrative argument concerning the likelihood of long term health impact is novel and has not been applied longitudinally to HiAP in any other setting. This study demonstrated that producing the evidence for HiAP’s impact called for in Leppo et al. [37] is possible with a longitudinal design and partnership between public servants and academics. Further, Guglielmin et al. [38] conducted a scoping review of local implementation of HiAP and concluded that contribution to theory development on HiAP implementation requires further research that ‘specifically investigates the facilitators and barriers of HiAP locally within their political and policy context’. This is precisely what our research has done by highlighting the ways in which the South Australian economic and political contexts had a significant impact on implementation and so influenced the likelihood of health outcomes.

Limitations to our research are that the attribution of causality is necessarily tentative and relies on imputed evidence. In the actions we observed, HiAP was a facilitator of change with many partners. This makes determining its contribution difficult. While the program logic shows a smooth flow through of action emphasising the contribution of the HiAP initiative, the actual picture is messier, with contributions, and in some cases the major contribution, coming from other sectors. However, interview respondents consistently informed us that in every case HiAP played a facilitating role and we were able to document that over time. There are also limits to how much account we could take of context. Labonté [39] noted in a commentary responding to our theory-based program logic that our work did not include ‘considerations of political and economic shifts at the national or global scales (eg, hyperglobalization, capital mobility, economic financialization, and tax competition)’ and that it ‘assumes that the policy actors at the South Australian state level have the capacities to implement HiAP in ways that can institutionalize sustained improvements in health equity’. Our program logic model was able to take into account some globalised context and did this, for example, in terms of the closure of an automotive manufacturing plant and the general impact of de-industrialisation in South Australia. It is, however, difficult to measure and account for the kind of global changes Labonté refers to in research which focuses on local activity. Institutional and political constraints meant SA HiAP focused on improving daily living conditions and was not able to address the underlying drivers of health inequities.

## Conclusions

We draw five key lessons from our evaluation of SA HiAP to assist other jurisdictions using or considering using the approach. These are:

1. Context is vital in shaping implementation. It both helped and hindered HiAP in a range of ways. Constant monitoring of the context was important to the implementation and evaluation of HiAP. Such careful attention helps the initiative to adapt to changing political and economic circumstances and helps evaluators make sense of why HiAP was adapting and how successfully it did so.
2. HiAP focused mainly on the implementation of initiatives that would change daily living conditions and did not challenge the basic structures of society which determine inequities. None of its actions were concerned with the SDH defined as changing the underlying distribution of power, money and resources [40]. This reflects several factors. SA HiAP was implemented at a level of government which does not control many aspects of the distribution of resources. It was also a project built on achieving a high degree of consensus (win-win strategies were favoured) and had very few avenues for citizen participation. Redistributive policies are likely to be contentious and require strong citizen involvement [21]. They will also require strong political leadership and policy development which includes advocacy for the health and social benefits of a more equal society. HiAP’s emphasis on consensus and win-win solutions may not always be appropriate when a strong advocacy approach is required, and so other approaches to intersectoral action for healthy public policy will still be required.
3. Regional government-based HiAP initiatives need to be complemented by those in national governments where the levers to change the distribution of power, money and resources are more powerful.
4. Not all the initiatives HiAP embarked on met with success. This is not surprising when the initiatives were dealing with many sectors with varied agendas in a context in which the public service was being subjected to considerable cutbacks. HiAP is an innovative approach at the cutting edge of the new public health and has to be allowed to experiment and sometimes fail.
5. HiAP was, in the main, successful at keeping the focus of projects on whole populations and universal policies and initiatives which then require relatively small shifts to have a significant impact across a population [41]. We found evidence of reversion to a lifestyle focus but this was very minor compared to the initiatives’ success in focussing on population-wide strategies, which is impressive when there are so many drivers to revert to behaviour-driven strategies. SA HiAP’s predominant focus on projects was recognised by the Health Department as limiting the sustainability and systemic effectiveness of HiAP. This has been addressed by it now being supported by a legislative mandate (for example, through PHPA) and a reduced focus on individual projects.

The SA HiAP approach had a small core budget. The approach was constrained in the extent to which it could address the underlying economic and power distribution impact of health, but can be judged to have had a minor impact on longer term population health. Many drivers of health are outside the remit of a state government. HiAP could do more to address health inequities and a promising option may be to partner with citizen groups and others to create a strong constituency for action on more structural determinants of health and a political will for intersectoral policy action for health and equity.

## Abbreviations

HiAP: Health in All Policies
HLA: Health lens analysis
PHPA: Public health partner authority
PLM: Program logic model
SA: South Australia
SASP: South Australian strategic plan
SDH: Social determinants of health
WHO: World Health Organization
